# Quantitative Evaluation of Postural SmartVest’s Multisensory Feedback for Affordable Smartphone-Based Post-Stroke Motor Rehabilitation

**DOI:** 10.3390/ijerph22071034

**Published:** 2025-06-28

**Authors:** Maria da Graca Campos Pimentel, Amanda Polin Pereira, Olibario Jose Machado Neto, Larissa Cardoso Zimmermann, Valeria Meirelles Carril Elui

**Affiliations:** 1 Instituto de Ciências Matemáticas e de Computação, Universidade de São Paulo (USP), São Carlos 13566-590, SP, Brazil; 2 Programa de Pós-Graduação Interunidades em Bioengenharia, EESC-FMRP-IQSC, Universidade de São Paulo (USP), Ribeirão Preto 14048-900, SP, Brazilvelui@fmrp.usp.br (V.M.C.E.)

**Keywords:** stroke rehabilitation, wearable technology, multisensory feedback, smartphone application, postural balance, mHealth, stabilization, digital intervention

## Abstract

Accessible tools for post-stroke motor rehabilitation are critically needed to promote recovery beyond clinical settings. This pilot study evaluated the impact of a posture correction intervention using the Postural SmartVest, a wearable device that delivers multisensory feedback via a smartphone app. Forty individuals with post-stroke hemiparesis participated in a single supervised session, during which each patient completed the same four-phase functional protocol: multidirectional walking, free walking toward a refrigerator, an upper-limb reaching and object-handling task, and walking back to the starting point. Under the supervision of their therapists, each patient performed the full protocol twice—first without feedback and then with feedback—which allowed within-subject comparisons across multiple metrics, including upright posture duration, number and frequency of posture-related events, and temporal distribution. Additional analyses explored associations with demographic and clinical variables and identified predictors through regression models. Wilcoxon signed-rank and Mann–Whitney U tests showed significant improvements with feedback, including an increase in upright posture time (p<0.001), an increase in the frequency of upright posture events (p<0.001), and a decrease in the total task time (p=0.038). No significant subgroup differences were found for age, sex, lateralization, or stroke chronicity. Regression models did not identify significant predictors of improvement.

## 1. Introduction

In 2021, Noncommunicable Diseases (NCDs), often referred to as chronic diseases, were responsible for at least 43 million deaths worldwide [1]. These enduring illnesses arise from a mix of genetic, physiological, environmental, and behavioral influences. The main categories of NCDs encompass cardiovascular diseases (including heart attacks and strokes), cancers, chronic respiratory conditions, and diabetes. Managing and treating NCDs is both costly and complex, which not only elevates healthcare expenses but also imposes substantial psychological stress on patients and their caregivers [1].

Recent studies have provided valuable insights into the management and prevention of noncommunicable diseases (NCDs), emphasizing the importance of integrated care and community-based interventions. The World Health Organization’s (WHO) Package of Essential Noncommunicable Disease Interventions for Primary Health Care outlines adaptable protocols for empowering primary care professionals and health workers in NCD management, particularly in low-resource settings. These interventions offer practical solutions to the global burden of NCDs by focusing on early detection and integrated management strategies within primary healthcare systems [2]. Among the many studies in this field, recent review articles highlight the potential of community pharmacies in expanding access to NCD services, especially in low- and middle-income countries (LMICs). Pharmacists, through improved education, screening, and management, have significantly enhanced disease control and patient satisfaction in these regions, addressing resource shortages and legal barriers that limit healthcare access [3]. Furthermore, emerging review research underscores the crucial role of inflammation in the pathogenesis of various NCDs, including obesity, diabetes, cardiovascular diseases (CVD), and cancer, thus offering a potential avenue for therapeutic interventions through dietary changes [4]. Periodontal diseases, as highlighted in the synthesis report by Herrera et al., have also been identified as key contributors to cardiovascular risk, with studies suggesting that the treatment of periodontitis can improve systemic health outcomes, particularly in relation to CVD and diabetes. Collaborative care involving oral health professionals and family doctors is essential for early detection and better management of these interconnected conditions [5]. These findings collectively contribute to a broader understanding of NCD prevention and management, highlighting the need for multidisciplinary approaches to improve patient outcomes and quality of life [6].

When viewed in this broader context, stroke, a significant NCD, has seen its impact grow from 1990 to 2021, with increasing contributions from various risk factors [7]. In 2021, there were approximately 7.25 million deaths due to stroke and 11.9 million new cases of stroke [8]. These figures result in millions of individuals experiencing permanent disabilities, thereby placing immense pressure on families and communities [9]. These realities underscore the critical need for effective, accessible, and affordable strategies to enhance stroke monitoring, prevention, emergency care, and rehabilitation globally. Implementing these measures is essential to mitigate the growing burden of stroke across all nations [7].

Among the various consequences of stroke, hemiparesis is one of the most widespread and devastating conditions [10]. For these individuals, mastering trunk control is essential for performing a range of functional activities [11]. Research indicates a strong link between maintaining proper posture and enhanced walking ability in patients undergoing acute stroke rehabilitation [12]. Unlike those without hemiparesis, who typically maintain symmetrical and balanced body alignment, individuals with hemiparesis often exhibit asymmetrical posture due to unilateral muscle weakness, resulting in imbalanced or tilted positions [13]. We define “best-at-the-time posture,” “optimal posture,” “target posture,” and “upright posture” as equivalent terms for the optimal trunk alignment achievable by hemiparetic patients during standing, within their motor limitations. Extensive efforts in the literature have focused on improving trunk stability [14], compensatory trunk movements [15], motor control [16], and mobility access [17] for stroke survivors. A systematic review has confirmed that trunk training significantly improves trunk control, sitting and standing balance, and overall mobility [18].

In response to this ongoing clinical challenge, therapeutic options in stroke rehabilitation have continued to evolve. For instance, Transcranial Ultrasound Stimulation has emerged as a promising non-invasive deep brain neuromodulation technique, which offers millimeter-accuracy spatial resolution and significant penetration depth, thereby establishing novel neurorehabilitation protocols [19]. Additionally, spinal cord stimulation has been identified as an effective intervention for inducing plasticity in the corticospinal tract, which leverages the largely intact spinal cord circuitry post-stroke to enhance motor recovery [20]. Alongside these approaches, there is a growing interest in alternative technologies. Examples include virtual reality and robotic solutions [21,22], visual biofeedback [23], as well as wearable technologies [24,25].

While these solutions hold promise, effective functional rehabilitation for stroke patients demands both body awareness and torso control to ensure upper limb functionality [11,14,26,27]. However, high costs and limited availability of traditional treatments [28,29,30,31] underscore the need for affordable alternatives. Although the number of mHealth smartphone apps for stroke patients is increasing [32], few specifically target trunk control [33,34,35]. Additionally, while intrinsic and extrinsic feedback is essential for motor learning post-stroke [36,37,38], and therapists invest significant time in providing this feedback [39,40], the integration of multisensory smartphone-based feedback for upper body rehabilitation remains limited [41]. Although smartphone-based multisensory feedback has proven effective for postural monitoring in healthy individuals [42], similar solutions for stroke patients are still lacking.

**Figure 1:**
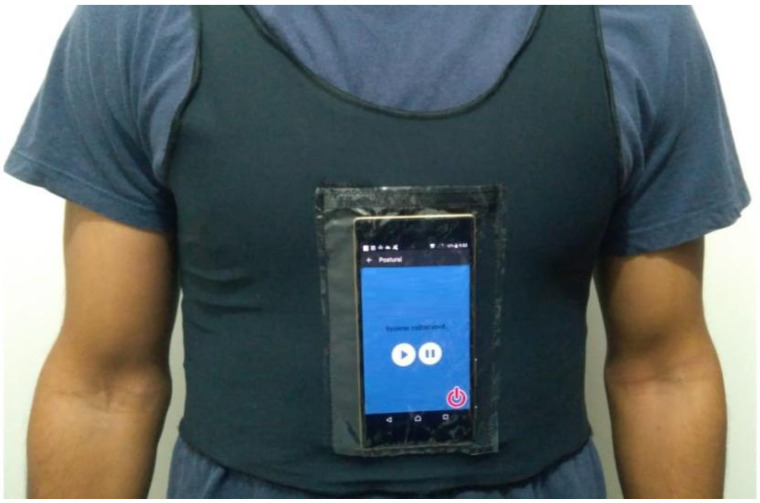
Postural SmartVest: athletic compression tank top with a smartphone pocket and a customizable Android app that delivers multisensory feedback [43]. The screen displays the message “System calibrated” and contains buttons to start, pause, and exit.

To bridge this gap, we developed Postural SmartVest (Figure 1), an affordable wearable technology that leverages low-cost smartphone resources by (1) continuously monitoring sagittal and frontal plane changes through built-in accelerometers and (2) providing visual, tactile, and auditory feedback to guide patients in achieving optimal posture [43]. Therefore, based on the monitoring of postural changes, the system provides feedback to guide the patient back to the correct posture. Our iterative design resulted in a modified athletic compression tank top with a secure smartphone pocket and a customizable Android app that delivers multisensory feedback. In validation studies, patients reported high ratings for weight, comfort, effectiveness, and ease of use, while therapists observed positive impacts on rehabilitation sessions and expressed willingness to recommend the device. Furthermore, the study identified a significant improvement in posture awareness when feedback was provided, compared to when no feedback was given, demonstrating the effectiveness of Postural SmartVest in enhancing rehabilitation outcomes [43].

To comprehensively evaluate the effectiveness of the Postural SmartVest, this study analyzes the data captured during two phases of an experimental session conducted with 40 patients: the baseline phase (without feedback) and the intervention phase (with feedback). The data from both phases were captured to allow for a comparison between the two.

We hypothesized that providing real-time multisensory feedback through the Postural SmartVest would lead to improved postural control and increased frequency of optimal postures during functional rehabilitation tasks. Specifically, we expected that patients would spend more time in the optimal posture and exhibit more corrective movements (leaning forward, backward, left, and right) in response to the feedback provided during the intervention phase compared to the baseline phase.

To test this hypothesis, we assessed the impact of the intervention by examining the distribution of postures, including optimal posture, as well as forward, backward, left, and right inclinations. These postural changes were quantified based on two primary dependent variables: (1) time spent in the optimal posture, which was measured in seconds; (2) the frequency of corrective movements, such as leaning forward, backward, left, and right, with each inclination being allowed for up to 5 s outside of the optimal position before feedback was triggered.

Additionally, we investigated whether the intervention’s effectiveness varied based on participant demographics and clinical characteristics, including gender, time since stroke onset, limb lateralization, and scores on the Functional Independence Measure (FIM) scale. The FIM scale, which ranges from 18 to 126, provides the classification of individuals by their ability to carry out daily activities either independently or with assistance. Higher scores indicate greater independence. Functional outcomes were specifically measured by these scores, assessing how much patients were able to perform the activities of daily living (ADLs) and their need for assistance in these tasks.

By comparing baseline and intervention data, this study aims to determine the extent to which multisensory feedback from Postural SmartVest enhances postural control and contributes to improved functional outcomes in stroke rehabilitation. Our findings demonstrate that Postural SmartVest effectively enhances postural control, thereby advancing rehabilitation outcomes for stroke patients.

## 2. Materials and Methods

### 2.1. Ethical Procedures

The study received prior approval from the Research Ethics Committee of the Ribeirão Preto School of Medicine, University of São Paulo, Brazil, under the CAAE (Certificate of Presentation for Ethical Consideration) 57234816.3.0000.5440 and Approval Code 1.850.318, dated 1 December 2016.

All participants provided written informed consent prior to their inclusion in the study, ensuring compliance with ethical guidelines for research involving human subjects. Participants in this study remained anonymous and could not be identified from any information included in this paper.

### 2.2. Study Design

This study adopts a within-subject experimental design. We hypothesized that providing real-time multisensory feedback through the Postural SmartVest would lead to improved postural control and an increased frequency of optimal postures during functional rehabilitation tasks. Specifically, we hypothesized that patients would spend more time in the optimal posture and exhibit more corrective movements (leaning forward, backward, left, and right) in response to the feedback provided during the intervention phase compared to the baseline phase.

To test this hypothesis, data were captured during both baseline (without feedback) and intervention (with feedback) phases. We assessed two main dependent variables: (1) time spent in the optimal posture (measured in seconds); (2) frequency of corrective movements, such as leaning in various directions. Statistical comparisons were made using the Wilcoxon signed-rank test and Cohen’s d for effect size estimation.

Because both conditions were conducted sequentially under an identical protocol, device configuration, and physical environment, any observed differences can be directly attributed to the feedback condition. This controlled setting enhances the internal validity of the comparisons. While the original study focused primarily on qualitative aspects of usability, the present work introduces new quantitative analyses to investigate posture event distributions and identify predictors of feedback-driven improvement.

The sample size of n=40 aligns with published recommendations for pilot studies investigating behavioral or rehabilitation interventions [44,45]. Although these guidelines primarily address between-subject designs, the within-subject format used here increases statistical power by reducing inter-individual variability, thereby rendering n=40 adequate for exploratory purposes.

Although therapists participated in the prior usability study (e.g., in interviews), they were not subjects in this analysis. In the present study, they acted solely in a supervisory role, assisting their regular patients during the rehabilitation session in which data collection took place.

To ensure robustness in our analysis, we employed non-parametric tests due to the non-normal distribution of the data. The Wilcoxon signed-rank test was selected to compare differences between the baseline and intervention phases, while Cohen’s d was used to calculate effect sizes, providing an estimate of the magnitude of the intervention’s impact. These statistical methods allow for a more accurate assessment of the feedback’s influence on postural control, given the small sample size and the within-subject design. By focusing on the frequency of corrective movements and time spent in optimal posture, we aimed to quantify the specific outcomes of the intervention and provide insights for future studies that may include a larger sample size and additional variables.

### 2.3. Participants

The study included 40 post-stroke patients (27 males, 67.5%) with a mean age of 56.5 years (SD = 12.2) (Table 1). Functional independence was assessed using the Functional Independence Measure (FIM), categorizing participants as independent (n = 39) or requiring moderate assistance (n = 1). Among the participants, 24 had right hemispheric lesions, 16 had left hemispheric lesions, 38 experienced ischemic strokes, and two suffered hemorrhagic strokes. The duration of their condition ranged from 6 months to 19 years, with the majority (n = 30) between one and four years post-stroke. Participants were recruited from those attending rehabilitation sessions at least twice a week for one month at the same center where data collection took place. Inclusion criteria included chronic hemiparesis resulting from stroke, age 18 years or older, independent walking ability, cognitive capacity for communication, and participation in rehabilitation sessions for at least one month [43].

The therapists involved in the study were the same professionals who regularly worked with the participants in rehabilitation. All therapists had training in physical therapy or occupational therapy and had at least one year of practice in stroke rehabilitation. They acted solely in a supervisory role during the sessions.

### 2.4. Instrumentation

To ensure consistency in data collection, a single smartphone model (Motorola G running Android version 5.1) was utilized for all sessions. The smartphone was integrated with the Postural SmartVest application (Figure 1), embedded in athletic compression tank tops during both baseline (without feedback) and intervention (with feedback) rehabilitation phases [43].

### 2.5. Session Procedure

**Figure 2:**
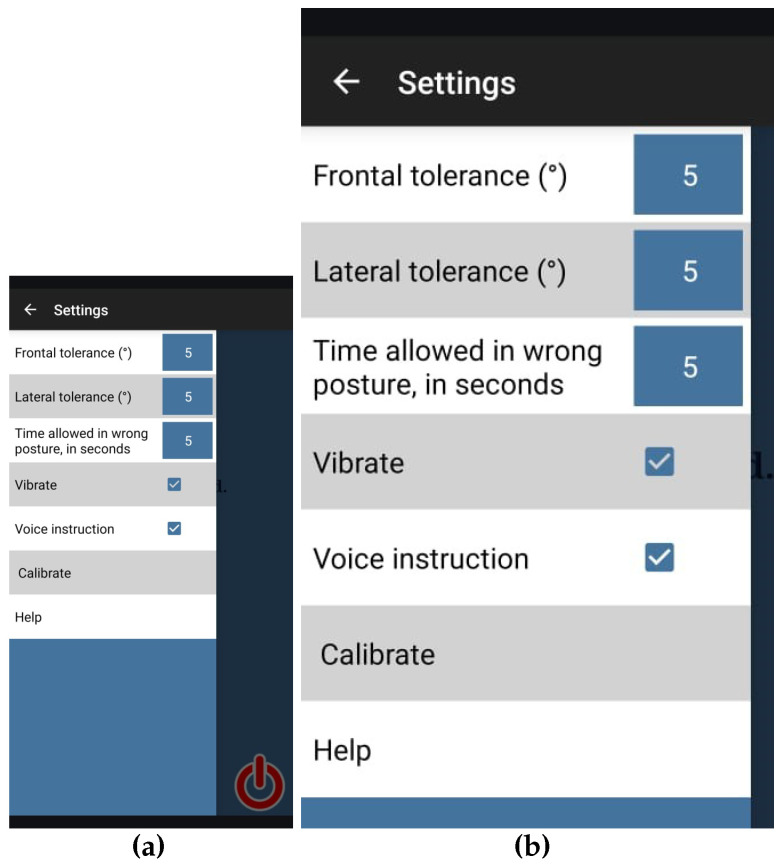
Postural SmartVest configuration panel: (**a**) full view and (**b**) close-up view [43].

Each session commenced with the therapist securing the compression tank top on the patient, calibrating the application to register the patient’s optimal standing posture at that moment, and initiating the monitoring process. The panel shown in Figure 2 was used for configuring the app. All patients had the application calibrated to their optimal standing posture. The configuration of other parameters was identical for all patients, as follows:

- Tolerance threshold angle for frontal movements: 5 degrees;
- Tolerance threshold angle for lateral movements: 5 degrees;
- Allowable duration for temporary deviations from the calibrated posture: 5 s.

Subsequently, each patient performed the protocol twice—first during the baseline phase, followed by the intervention phase—in consecutive sessions, each with an estimated duration of 10 min. In the baseline phase, the application did not provide feedback, but it recorded all movements. During the intervention phase, the application provided feedback through screen color changes (green and red), vibrations, and audio guidance.

### 2.6. Session Protocol

**Figure 3:**
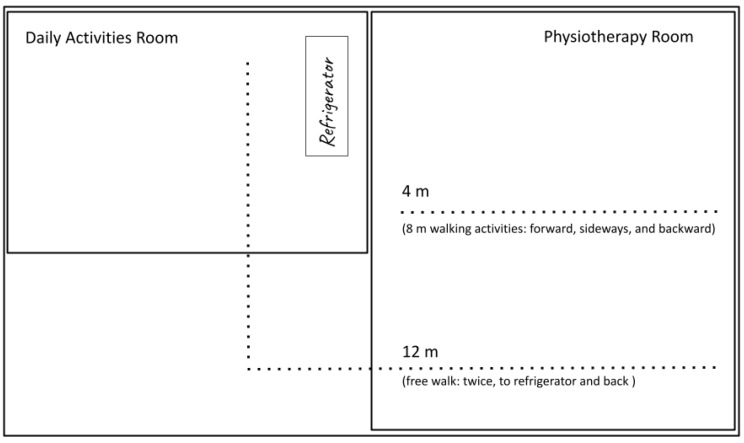
Therapy room layout [43].

The data collection protocol was consistent with the activities patients regularly perform during their rehabilitation sessions, which were conducted in the same room (Figure 3). Each patient had performed each activity at least three times within the past 30 days.

**Figure 4:**
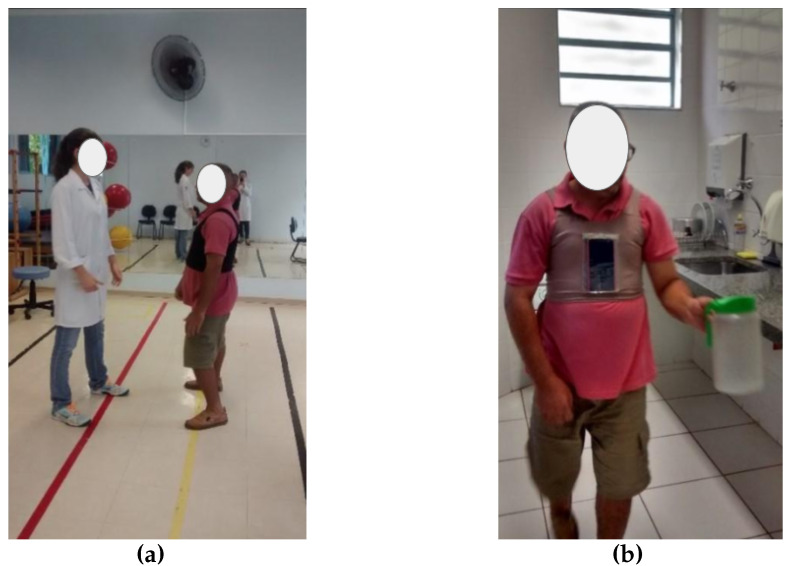
Examples of activities: (**a**) Ambulant activity and (**b**) upper limb activity [43].

Each patient followed a protocol comprising ambulatory-based daily activities conducted in the therapy room (Figure 4). The protocol consisted of four distinct phases:

1. The participant walked eight meters, incorporating forward, sideways, and backward walking.
2. They proceeded with a 12 m free walk to a refrigerator.
3. The participant engaged in an upper limb activity that entailed opening the refrigerator, taking a jar, and returning it to the fridge.
4. They completed a 12 m free walk back to the starting point.

### 2.7. Data Analysis

All statistical analyses were performed using Python version 3.11.11 on an Ubuntu 22.04.3 LTS (Jammy Jellyfish) operating system within the Google Colab environment, utilizing a CPU-only runtime without access to GPU or TPU accelerators. Data were analyzed using Python scripts running on Google Colab to assess the impact of the intervention by investigating posture distributions and patient demographics during baseline and intervention phases.

In addition to descriptive analyses, various statistical tests were employed to evaluate differences in posture distributions between the baseline and intervention phases. First, the normality of the data was assessed using the Shapiro–Wilk test (SW). For paired comparisons, if the data were normally distributed, the paired *t*-test (*t*) was applied; otherwise, the Wilcoxon signed-rank test (*W*) was used. Differences in proportions were examined using the chi-square test ($\chi^{2}$). Furthermore, subgroup comparisons among independent groups were conducted using the Mann–Whitney U test (*U*) for two-group comparisons and the Kruskal–Wallis test (*H*) for comparisons involving more than two groups. To quantify the magnitude of observed effects, Cohen’s d (*d*) was calculated for significant comparisons. These tests were selected and applied as appropriate to ensure robust and valid statistical inferences regarding the intervention’s impact.

Multiple linear regression analyses were also conducted to investigate whether demographic and clinical variables predicted improvements in posture during the intervention.

Clinical characteristics, stroke onset duration, limb lateralization, and Functional Independence Measure (FIM) scores were collected via structured pre-session interviews with patients. A trained therapist from the research team (second author) administered the FIM using the standardized protocol. These interviews were conducted individually at scheduled times immediately after participants completed the consent forms.

Postural distribution data were obtained during the session’s calibration phase, where the supervising therapist recorded each patient’s optimal upright posture through the application. These calibrated values served as the reference baseline for detecting postural deviations in subsequent analyses.

## 3. Results

### 3.1. Patients (n = 40) Characteristics

The participants had a mean age of 56.48 years (median = 57, SD = 12.25). Age was normally distributed, as confirmed by the Shapiro-Wilk test (SW = 0.95, *p* > 0.05).

**Figure 5:**
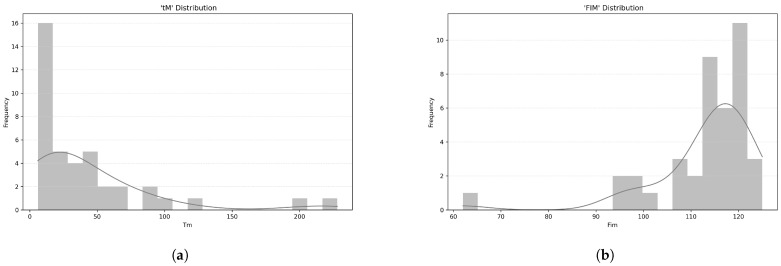
Patients’ characteristics: (**a**) Histogram of time since stroke in months (tM). (**b**) Histogram of Functional Independence Measure (FIM) scores.

The time since stroke onset (tM) averaged 44 months (median = 24, SD = 48.9), with a non-normal distribution, as indicated by the Shapiro-Wilk test (SW = 0.70, *p* < 0.001) (Figure 5a).

Functional Independence Measure (FIM) scores had a mean of 113 (median = 115.5, SD = 11.2), which are also not normally distributed, as shown by the Shapiro-Wilk test (SW = 0.72, *p* < 0.001) (Figure 5b). The mean FIM score (within the 18–126 range) of 113.00 (SD = 11.20) suggests that, on average, participants had a high level of functional independence. This means they were able to perform most daily activities with little or no assistance.

Regarding subgroup comparisons by gender, no statistically significant difference in age was found, as shown by the paired *t*-test (*t* = 0.06, *p* > 0.05).

Similarly, stroke duration did not differ significantly between genders, as indicated by the Mann–Whitney U test (*U* = 173, *p* > 0.05).

For FIM scores, the Mann–Whitney U test also revealed no significant difference between genders (*U* = 184, *p* > 0.05).

For affected limb laterality, the chi-square test for independence was used to assess the association with gender. The chi-square test found no significant association between the two variables ($\chi^{2}$ = 0.49, *p* > 0.05).

In summary, these findings suggest a relatively homogeneous sample in terms of demographic and clinical factors relevant to postural deviation analysis.

### 3.2. Postural Deviation Analysis

**Figure 6:**
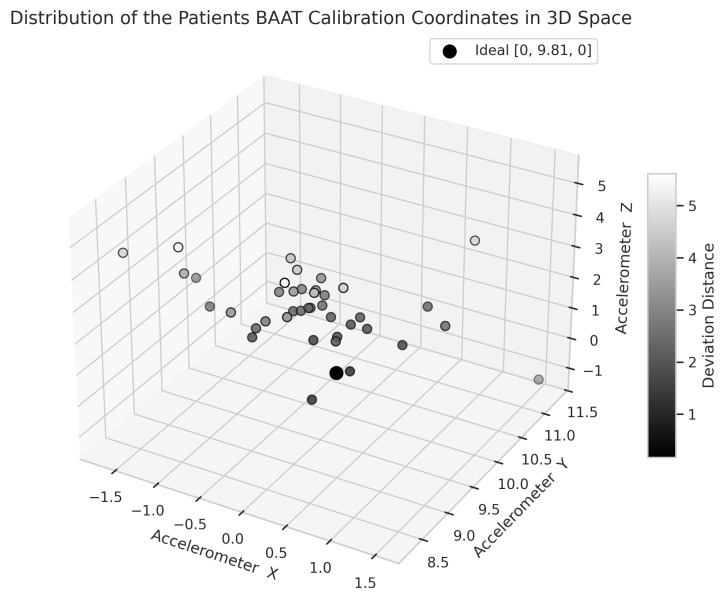
Three-dimensional visualization of patients’ best-at-the-time (BAAT) posture and the ideal reference point.

The Euclidean distance is a measure of the straight-line distance between two points in space. We use it to calculate the difference between each patient’s calibration coordinate, representing the best posture the patient can hold at the time, as assessed by the therapist based on the patient’s current capabilities and condition, and the ideal reference point, as illustrated in Figure 6.

**Figure 7:**
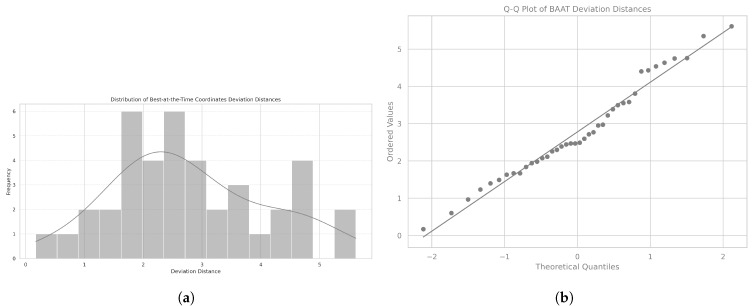
Distribution of patients’ deviation distances. (**a**) Histogram of Euclidean distances between each patient’s best-at-the-time (BAAT) and the ideal reference point. (**b**) Q-Q plot of the deviation distances relative to the BAAT posture.

The mean Euclidean distance was 2.78, with a standard deviation of 1.31, and the distribution followed a normal distribution, as confirmed by the Shapiro-Wilk test (SW = 0.97, *p* = 0.432). The corresponding histogram is shown in Figure 7a, and the Q-Q plot assessing normality is displayed in Figure 7b.

When analyzed by gender, data from both male patients (mean = 2.56, SD = 1.29) and female patients (mean = 3.24, SD = 1.37) showed normal distributions, as confirmed by the Shapiro-Wilk test ($SW_{\mathrm{male}}$ = 0.96, *p* = 0.367; $SW_{\mathrm{female}}$ = 0.89, *p* = 0.082).

No significant difference was found between genders in the Euclidean distance, with the statistical comparison showing a paired *t*-test value of −1.59 and a *p* value of 0.121, indicating no substantial variation between male and female patients.

Overall, the Euclidean distance between the patients’ calibration coordinates and the ideal reference point followed a normal distribution, with no significant differences observed between genders. These findings provide a baseline for further investigation into factors influencing postural alignment and deviation.

### 3.3. Baseline vs. Intervention: Total Time Spent in Each Phase

During the baseline phase, patients spent an average of 9.10 min (median = 7.61, SD = 5.21). The data did not follow a normal distribution, as confirmed by the Shapiro-Wilk test (SW = 0.83, *p* = 0.003).

In the intervention phase, patients spent an average of 7.75 min (median = 7.08, SD = 5.20). The distribution again was not normal, as confirmed by the Shapiro-Wilk test (SW = 0.96, *p* = 0.140).

**Figure 8:**
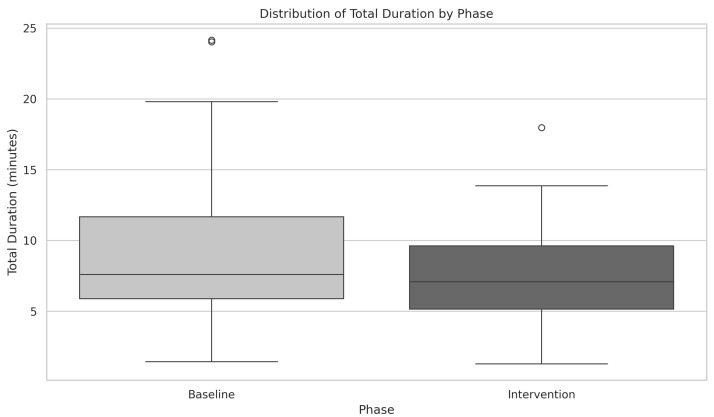
Comparison of total duration spent in baseline and intervention phases. Wilcoxon signed-rank test *W* = 255.5, *p* = 0.038, with Cohen’s *d* = 0.31, indicating a statistically significant difference with a small effect size.

A significant difference between the time spent in the baseline and intervention phases was found using the Wilcoxon signed-rank test (*W* = 255.5, *p* = 0.038) as indicated in Figure 8. The corresponding Cohen’s *d* = 0.31 suggests a small effect size, indicating that while the difference between baseline and intervention is statistically significant, the practical impact of this difference may be modest.

### 3.4. Baseline vs. Intervention: Impact on the Number of Posture-Related Events per Patient

The total number of posture-related events during both phases was 3535 (baseline = 1240, intervention = 2295). We consider five event types (or movement types) for all patients:

- “User is up straight” the best-of-the-time posture the patient can hold at the time, as assessed by the therapist, based on the patient’s current capabilities and condition;
- “User leaned back” associated with trunk extension (backwards);
- “User leaned forward” associated with trunk flexion (forward);
- “User leaned to the left” associated with left-side bending;
- “User leaned to the right” associated with right-side bending.

In absolute terms, the descriptive statistics for each event type during the baseline phase were as follows: for “User is up straight,” total 212 (mean = 5.30, SD = 3.95); for “User leaned back,” total 261 (mean = 6.53, SD = 15.67); for “User leaned forward,” total 582 (mean = 14.55, SD = 18.34); for “User leaned to the left,” total 101 (mean = 2.53, SD = 6.58); and for “User leaned to the right,” total 84 (mean = 2.10, SD = 4.25). Shapiro-Wilk tests revealed that all baseline distributions deviated significantly from normality (all with *p* < 0.001).

During the intervention phase, descriptive statistics showed the following: for “User is up straight,” total 550 (mean = 13.65, SD = 7.23); for “User leaned back,” total 162 (mean = 4.05, SD = 4.39); for “User leaned forward,” total 962 (mean = 24.05, SD = 22.88); for “User leaned to the left,” total 338 (mean = 8.45, SD = 12.70); and for “User leaned to the right,” total 283 (mean = 7.08, SD = 7.50). Again, Shapiro-Wilk tests indicated significant deviations from normality for all event types (all with *p* < 0.001).

**Figure 9:**
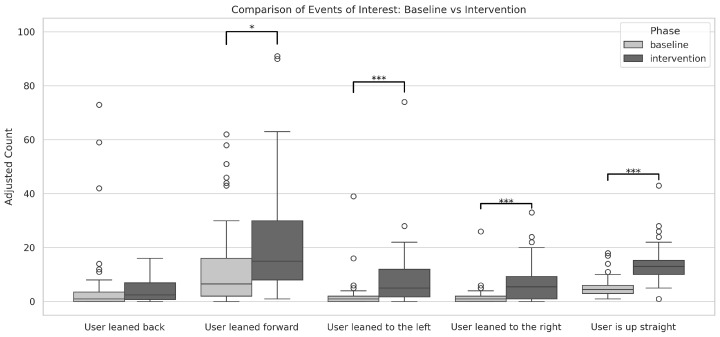
Comparison of number of posture-related events between baseline and intervention phases. Significant improvements were observed in “User is up straight” events, with a very large effect (Wilcoxon signed-rank test *W* = 13, *p* < 0.001; *d* = −1.45), as well as in “User leaned to the left” (*W* = 8, *p* < 0.001), “User leaned to the right” (*W* = 57, *p* < 0.001), and “User leaned forward” (*W* = 199.5, *p* < 0.05), all showing moderate to large effects. No significant difference was found in “User leaned back” events (*W* = 148, *p* > 0.05). Asterisks indicate statistical significance: * *p* < 0.05, *** *p* < 0.001.

A within-subjects Wilcoxon signed-rank test was then conducted to compare the baseline and intervention phases for each event type, yielding the following results: for “User is up straight,” *W* = 13, *p* < 0.001; for “User leaned back,” *W* = 148, *p* > 0.05; for “User leaned forward,” *W* = 199.5, *p* < 0.05; for “User leaned to the left,” *W* = 8, *p* < 0.001; and for “User leaned to the right,” *W* = 57, *p* < 0.001 (Figure 9).

Among the observed events, the effect sizes measured by Cohen’s *d* were as follows: for “User leaned back,” *d* = 0.24 (not statistically significant); for “User leaned forward,” *d* = −0.45; for “User leaned to the left,” *d* = −0.57; for “User leaned to the right,” *d* = −0.80; and for “User is up straight,” *d* = −1.45. These results indicate that, aside from “User leaned back,” which showed a negligible effect, the other events exhibited effects ranging from small to very large, with “User is up straight” demonstrating a very large effect size.

Importantly, the count for the event type “User is up straight” showed a statistically significant difference between phases and a very large effect size. This finding extends the results from our design and usability study, which was limited to the event count [43], further corroborating that the intervention successfully increased the frequency of events in which patients maintained the best-at-the-time posture.

Moreover, comparisons across other movement types revealed that the intervention also had a measurable impact on lateral movements (both to the left and right) as well as forward-inclining movements. These results suggest that the intervention not only enhanced patients’ ability to achieve the best-at-the-time posture but also led to a higher frequency of corrective movements, emphasizing the effectiveness of the guiding feedback in promoting motor corrections and overall postural improvement during the intervention phase.

### 3.5. Baseline vs. Intervention: Analysis of Mean Frequencies and Temporal Distribution of Best-at-the-Time Posture Events

**Figure 10:**
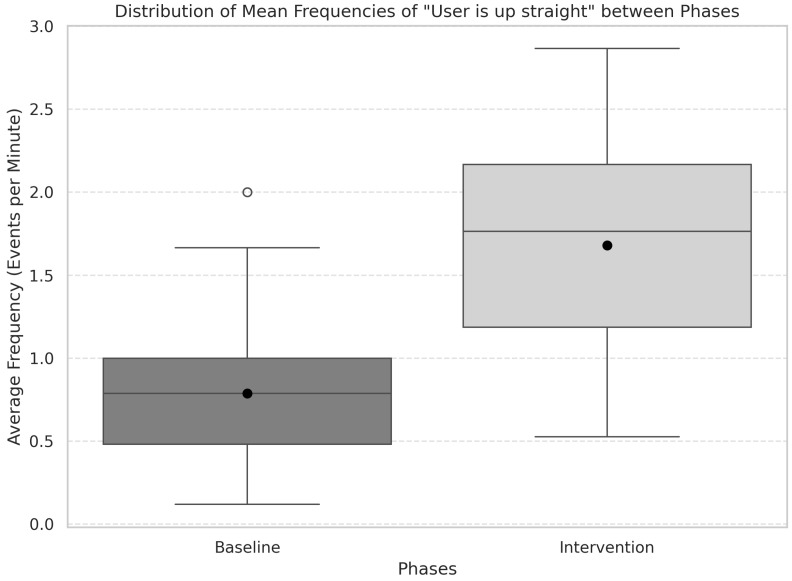
Comparison of mean frequency of “User is up straight” events per minute in baseline and intervention phases. Wilcoxon signed-rank test (*W* = 28, *p* < 0.001) indicates a significant improvement in posture maintenance with a very large effect size (Cohen’s *d* = −1.71).

An analysis of the frequency of “User is up straight” events serves as an indicator of patients maintaining correct posture across both the baseline and intervention phases. The analysis compares these phases by examining the mean frequency (events per minute) of these posture events (Figure 10). The distribution for baseline was verified to deviate from normality, as confirmed by the Shapiro-Wilk test (SW = 0.90, *p* = 0.002). A significant difference was observed between baseline and intervention, as shown by the Wilcoxon signed-rank test (*W* = 28, *p* < 0.001). Moreover, Cohen’s *d* yielded −1.71. This increase in mean frequency suggests a consistent improvement in patients’ posture, supporting the effectiveness of the intervention in promoting correct posture, while the very large effect size indicated by Cohen’s *d* underscores the practical significance of the observed improvement.

**Figure 11:**
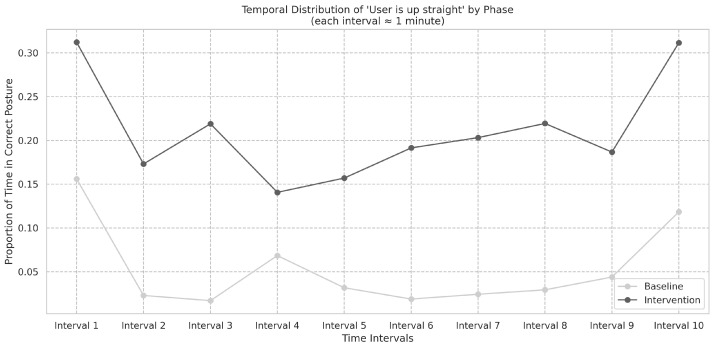
Temporal distribution of ”User is up straight” events. Wilcoxon signed-rank test *W* = 46, *p* < 0.001, with Cohen’s *d* = −1.40, indicating a large effect size and improved posture maintenance.

Figure 11 summarizes the frequencies in the baseline and intervention phases. The temporal distribution of “User is up straight” events in the baseline phase was assessed using the Shapiro-Wilk test (*S* = 0.62, *p* < 0.001) and did not follow a normal distribution. The difference in frequencies between the baseline and intervention phases was found to be significant, as shown by the Wilcoxon signed-rank test (*W* = 46, *p* < 0.001), and Cohen’s *d* was −1.40.

These results suggest that the intervention produced a robust improvement in patients’ ability to maintain correct posture, as evidenced by the significant increase in the frequency of “User is up straight” events during the intervention phase. The very large effect size indicated by Cohen’s *d* further underscores the practical significance of this improvement.

### 3.6. Baseline vs. Intervention: Total Time and Demographics

We hypothesized that the intervention would lead to a significant change in total time spent in the optimal posture during the intervention phase compared to the baseline phase. As a follow-up, we aimed to investigate whether these changes in total time would also be influenced by gender, age group, affected hemisphere, and time since stroke. Subgroup analyses were performed to assess if the impact of the intervention—measured as the difference in total time between the intervention and baseline phases—varied across patient demographics.

No significant differences were found across the subgroups, with all *p*-values greater than 0.05. Specifically, the Mann–Whitney U test was applied to gender (*U* = 172, *p* > 0.05), while the Kruskal–Wallis test was used to analyze age groups (*H* = 1.40, *p* > 0.05), affected hemisphere (*U* = 153, *p* > 0.05), and post-stroke time ranges (0–12, 13–24, 25–36, >36 months) (*H* = 1.92, *p* > 0.05).

Overall, these findings indicate that they did not vary significantly by gender, age group, stroke laterality, or post-stroke time.

### 3.7. Baseline vs. Intervention: Number of Best-at-the-Time Posture and Demographics

To assess whether the impact of the intervention—measured as the difference in the number of best-at-the-time postures achieved by patients between the baseline and intervention phases—varied across patient demographics, we conducted subgroup analyses. No significant differences were found, with all *p*-values greater than 0.05: for gender, Mann–Whitney U test (*U* = 196.5), *p* > 0.05; for age in the ranges <40, 40–60, >60, Kruskal–Wallis test (*H* = 2.36), *p* > 0.05; for affected hemisphere, Mann–Whitney U test (*U* = 167), *p* > 0.05; and for post-stroke time in the ranges 0–12, 13–24, 25–36, and >36 months, Kruskal–Wallis test (*H* = 4.63), *p* > 0.05.

These results suggest that the intervention’s impact on the number of best-at-the-time postures achieved by patients did not vary significantly with gender, age group, stroke laterality, or post-stroke time.

### 3.8. Regression Analyses

To further explore predictors of positive postural outcomes, we conducted multiple linear regression analyses using two separate outcome measures: (1) the total time (in minutes) that patients maintained the “User is up straight” posture; (2) the number of occurrences of “User is up straight.” Both analyses were aimed at assessing whether demographic and clinical variables—namely, age, gender, stroke laterality, and time post-stroke—were associated with greater improvements during the intervention phase relative to baseline. Moreover, a standard multiple linear regression analysis further evaluated whether the Euclidean distance from the ideal position was associated with postural improvement.

In the total time-based analysis, an OLS regression with robust standard errors ($R^{2}=0.145$) did not identify statistically significant effects of the predictors. Similarly, demographic factors did not significantly predict the magnitude of this improvement. However, the regression analysis based on the number of occurrences indicated an overall increase in the desired events during the intervention (pseudo $R^{2}=0.067$, $R^{2}=0.43$).

Moreover, a multiple linear regression analysis revealed that the Euclidean distance from the ideal position (β=−0.76,p>0.05) was not a significant predictor of the difference in the number of correct positions between the baseline and intervention phases ($R^{2}=0.06,F\left(5,34\right)=0.50,p>0.05$).

These findings suggest that, while the intervention positively impacted patients’ ability to achieve the correct posture, the specific demographic and clinical variables examined do not robustly predict the degree of improvement. Additionally, the Euclidean distance from the ideal position was not a significant predictor of postural correction, indicating that initial postural deviation alone does not determine the extent to which patients benefit from the intervention.

Although no significant predictors were identified in the regression analysis, this does not discredit the effectiveness of the intervention. Other factors, such as intervention intensity, individual differences, and psychological aspects, may have played a role in the outcomes. Additionally, the relatively small sample size could have affected the statistical power, potentially limiting our ability to detect significant predictors.

## 4. Discussion

Healthcare literature highlights the potential of smartphones [17,46] and wearables [47,48,49,50] in empowering individuals, aiding diagnoses, promoting behavior change, and enabling self-monitoring. Wearable and rehabilitation devices for the head, limbs, and torso enhance training outcomes through valuable feedback [41,51,52,53]. Numerous studies validate inertial sensors for balance and gait assessment [33,54,55,56], supported by comprehensive reviews [57,58], even in chronic stroke [59]. Research on wearable technology for poststroke support includes step activity monitors [60,61], intelligent insoles for gait analysis in hemiparesis [62], and home-based smartwatch rehabilitation systems for remote monitoring [63]. Additional studies evaluate haptic nudging via wrist devices on upper limb movements during inpatient rehabilitation [64] and smartphone-based kinematic assessments in chronic stroke survivors [17]. Literature also explores wearable solutions for postural monitoring, such as rhythmic haptic cueing for gait in hemiparesis [65], Smart Pose using smartphone sensors to correct neck posture [66], 3-axis accelerometer biofeedback for neck angle reduction [56], elastic t-shirts with posture sensors [67], spine posture monitoring devices [68], and posture differentiation systems [69].

In previous work, we presented the design and validation of an affordable and simple wearable smartphone-based multisensory feedback system for torso posture correction, utilizing an off-the-shelf Android smartphone running a dedicated application, integrated into a fitness top worn on the patient’s chest [43]. The mobile application provides instantaneous multisensory feedback on the patient’s posture by leveraging accelerometer data, which is calibrated against the patient’s initial target posture to deliver real-time corrective guidance.

This study analyzed data collected in a within-subject investigation with 40 post-stroke patients, assessing their ability to achieve optimal posture during rehabilitation sessions. The results demonstrated that the intervention significantly increased the frequency of optimal postures, reinforcing its potential as a practical and cost-effective rehabilitation tool. However, several findings warrant further discussion.

### 4.1. Impact of the Intervention on Postural Outcomes

The intervention significantly improved the number of instances in which patients maintained the best-at-the-time posture, confirming its meaningful impact on postural behavior. This result is consistent with the findings of our previous study.

However, the present study provides a more in-depth analysis, showing that, beyond an increase in the number of target postures achieved, the difference between phases had a very large effect size. Moreover, the increased frequency of correct postures was sustained throughout the intervention, also presenting a large effect size, reinforcing the role of real-time feedback in promoting motor learning.

Additionally, the results revealed that, besides improving the number of correct postures, the number of movements to the left, right, and forward also increased significantly. This suggests that the feedback provided by the system not only helps patients reach the target posture but also guides them in correcting postural deviations dynamically.

Furthermore, patients spent less time in the intervention phase compared to the baseline. On one hand, although this difference was statistically significant, its practical impact appears to be modest. On the other hand, considering that not only was the number of target postures higher but also the total number of movements, thus achieving these improvements in a shorter time suggests that the intervention contributed to a more efficient motor response. This efficiency gain may reflect an enhanced ability to correct posture with fewer adjustments, indicating that patients adapted more effectively to the feedback provided.

Our previous validation study reported high patient ratings for weight, comfort, effectiveness, and ease of use, while therapists noted positive impacts on rehabilitation sessions and expressed a strong willingness to recommend the device [43]. These findings reinforce that the efficiency in postural correction observed in the present study was achieved in a way that was well received by both patients and clinicians, further supporting the feasibility of this low-cost intervention for broader clinical implementation.

### 4.2. Effectiveness Across Subgroups and the Role of Initial Postural Deviation

The regression analyses revealed that demographic and clinical variables, including age, gender, stroke laterality, and time post-stroke, did not significantly predict the degree of improvement in postural outcomes. This suggests that the intervention was effective across different patient subgroups, independent of these individual characteristics. These findings align with previous research indicating that real-time biofeedback can facilitate motor relearning irrespective of patient demographics.

Additionally, an analysis of initial postural deviation, measured via Euclidean distance from the ideal posture, showed that this factor was not a significant predictor of improvement. This suggests that initial misalignment alone does not determine the extent to which patients benefit from the intervention. Instead, the absence of a significant association with initial postural deviation suggests that factors beyond static alignment, such as motor control adaptability, proprioceptive feedback, and patient engagement, may influence rehabilitation outcomes.

### 4.3. Clinical and Practical Implications

The Postural SmartVest offers continuous posture monitoring, providing ongoing feedback to patients, which can be beneficial both in rehabilitation and home environments—a feature highlighted by therapists and patients in our usability study. Moreover, by leveraging commonly available smartphones, this approach is not only cost-effective but also accessible to a broader patient population, complementing traditional rehabilitation methods, particularly in low-resource environments where specialized equipment may be limited.

The current findings underscore the feasibility of using an off-the-shelf smartphone to deliver real-time postural feedback as part of a rehabilitation protocol. This quantitative analysis adds valuable insight by objectively measuring the effects of the intervention on postural outcomes, such as the increase in the duration of upright posture and the frequency of optimal posture events, demonstrating the tangible impact of this approach.

The lack of demographic-based differences in outcomes suggests that this intervention could be broadly applicable to diverse patient populations. Additionally, the absence of a predictive relationship between initial postural deviation and improvement highlights the importance of individualized rehabilitation strategies rather than relying on static baseline assessments.

### 4.4. Limitations and Future Directions

Several limitations should be considered when interpreting these findings. First, while the intervention demonstrated efficacy in improving postural outcomes, the study did not assess long-term retention or functional carryover beyond the intervention period. Future research should investigate whether improvements persist over time and whether they translate into functional benefits in daily activities.

Additionally, factors such as patient engagement, motivation, and cognitive function were not explicitly analyzed, yet they may contribute to individual differences in response to the intervention. Future studies should incorporate these variables to gain a more comprehensive understanding of patient-specific rehabilitation trajectories.

Another limitation of the current study is the underrepresentation of participants in the 20–43 age range. The demographic breakdown shows that only a small percentage of participants (7.5%) were in this age group, which may limit the generalizability of the findings to younger patients. While age is one of the most important risk factors for stroke, with 75% of strokes occurring in individuals aged 65 years and older [70], future research should aim to include a more balanced distribution of participants across all age ranges to better understand how the intervention may benefit patients of different ages.

Future studies should explore the long-term effects of the intervention and investigate its effectiveness across different settings, including both rehabilitation and home environments. Moreover, the intervention’s impact on various subgroups, such as gender, age, and stroke laterality, should be further examined, as the current study did not show significant differences across these groups. Additionally, other unmeasured variables, such as intervention intensity, individual differences, and psychological factors, may play a role in the outcomes. Expanding studies to include diverse patient characteristics and settings will provide deeper insights into the long-term applicability and real-world potential of the Postural SmartVest.

## 5. Conclusions

This study provides evidence supporting the use of the Postural SmartVest, a low-cost, smartphone-based multisensory feedback system, to improve postural alignment in post-stroke patients. The intervention resulted in significant improvements across multiple metrics, including a decrease in the total time spent performing the four phases of ambulatory-based daily activities, compared to the baseline (Wilcoxon signed-rank test *W* = 255.5, *p* = 0.038, with Cohen’s *d* = 0.31), which indicate a small effect size.

Additionally, the intervention led to significant improvements in posture-related events. Specifically, the frequency of achieving the target posture, indicated by the number of “User is up straight” events, increased significantly (Wilcoxon signed-rank test *W* = 13, *p* < 0.001), with a very large effect size (*d* = −1.45). Other posture-related movements, such as leaning to the left, right, and forward, also showed moderate to large effects, suggesting that the intervention not only enhanced patients’ ability to achieve the best-at-the-time posture but also led to a higher frequency of corrective movements to guide patients back to the optimal position.

The analysis of the mean frequency of “User is up straight” events per minute confirmed a significant improvement in posture maintenance during the intervention (Wilcoxon signed-rank test *W* = 28, *p* < 0.001), with a very large effect size (Cohen’s *d* = −1.71). Furthermore, the temporal distribution of these events revealed significant improvements in posture maintenance throughout the intervention period, as indicated by the Wilcoxon signed-rank test (*W* = 46, *p* < 0.001), with Cohen’s *d* = −1.40, which highlights the effectiveness of the feedback in promoting sustained improvements in posture.

The regression analyses revealed that, while the intervention led to significant improvements in posture maintenance, the examined demographic and clinical variables (age, gender, stroke laterality, and time post-stroke) did not significantly predict the degree of improvement. Specifically, the regression analysis based on the frequency of “User is up straight” events demonstrated a positive effect, with a significant increase in the occurrence of these desired postural events during the intervention (pseudo $R^{2}=0.067$, $R^{2}=0.43$). This suggests that the intervention effectively enhanced postural outcomes. However, the total time spent in the correct posture did not show statistically significant associations with the predictors (OLS regression $R^{2}=0.15$), and the Euclidean distance from the ideal posture was not a significant predictor of improvement in posture during the intervention. These findings suggest that while the intervention positively impacted posture, other unmeasured factors, such as intervention intensity, individual variability, and psychological factors, may have also influenced the results. The small sample size may have limited the power to detect these effects fully.

These results suggest that the Postural SmartVest is an effective tool for improving postural control in rehabilitation settings, as demonstrated by significant improvements in posture duration, event frequency, and overall posture maintenance. The intervention’s impact was consistent across various posture types and phases, with no significant differences observed between diverse patient groups, thus underscoring its potential for broad applicability in stroke rehabilitation. Future studies should explore the long-term effects of this intervention and assess its effectiveness across different settings, including both rehabilitation environments and home settings, where its impact on daily functioning and postural control could be further examined.

## Figures and Tables

**Table 1:** Participant characteristics as previously reported in the SmartVest validation study [43].

Patients Characteristic	n	%
**Patients** (n = 40)		
Male	27	67.5
Female	13	32.5
Age (years)		
20–31	2	5
32–43	1	2.5
44–55	15	37.5
56–67	15	37.5
68–80	7	17.5
years: mean 56.5, median 57, SD 48.7, min 20, max 78		
Affected hemisphere		
Left	16	40
Right	24	60
Stroke type		
Hemorrhagic	2	5
Ischemic	38	95
Time since stroke (months)		
0–12	16	40
13–24	5	12.5
25–36	4	1
37–48	5	12.5
48+	10	25
months: mean 44, median 24, SD 48.7, min 6, max 228		
Functional Independence (FIM)		
Independent	39	97.5
Moderate Assistance	1	2.5
score: mean 113, median 115.5, SD 11.2, min 62, max 125		

## Data Availability

The data presented in this study are available on request from the first author. The data are not publicly available due to ethical restrictions and other studies in progress.

## Abbreviations

The following abbreviations are used in this manuscript:

CVD	cardiovascular diseases
FIM	Functional independence measure
NCD	Noncommunicable Diseases
